# Pellagra Increasing

**Published:** 1916-02

**Authors:** 


### Pellagra Increasing.

The South Texas District Medical Association is alarmed over the growing prevalence of pellagra throughout East and South Texas. Despite the fight that has been constantly waged on this insidious disease since it made its first appearance in the State a few years ago, it has continued to increase, especially in the country communities and in small towns where sanitary conditions are poor. The medical fraternity is not agreed yet as to the cause of the disease, and a serious effort is to be made to definitely ascertain what causes it. A general campaign is to be conducted by the association to educate the people as to public health matters generally, and this can but result in good.

